# Modeling Human Airway Epithelial Barrier Penetration Using Birch Bet v 1 and Alder Aln g 1 Pollen Allergens During Sensitization Process

**DOI:** 10.3390/ijms26115169

**Published:** 2025-05-28

**Authors:** Daria N. Melnikova, Andrey E. Potapov, Tatiana V. Ovchinnikova, Ivan V. Bogdanov

**Affiliations:** 1 M.M. Shemyakin & Yu.A. Ovchinnikov Institute of Bioorganic Chemistry, The Russian Academy of Sciences, 117997 Moscow, Russia; cool.goyan@yandex.ru (A.E.P.); ovch@ibch.ru (T.V.O.); contraton@mail.ru (I.V.B.); 2 Moscow Center for Advanced Studies, 123592 Moscow, Russia

**Keywords:** allergy, sensitization, plant PR-10 proteins, pollen allergen, alder, Aln g 1, Bet v 1, Calu-3 cells, surfactant, airway epithelium

## Abstract

Pollen allergy is rated as a major public health problem, causing significant morbidity and adversely affecting the quality of people’s lives. The airway epithelium serves as the first line of defense in the respiratory system, playing a crucial role in orchestrating immune responses to allergens. In this work, we studied the important transport steps in the major alder pollen allergen Aln g 1 through the human airway epithelium in comparison with those of the birch pollen allergen Bet v 1. Using fluorescence spectroscopy, we showed that both allergens can destroy liposomes with a composition modeling the adult human pulmonary surfactant. Using a polarized Calu-3 monolayer, we showed similar efficiencies of Aln g 1 and Bet v 1 transport through the artificial epithelial barrier. Using qPCR, we showed that Aln g 1 upregulates the expression of IL-33, TSLP, IL-1β, CXCL8 in epithelial cells, playing an important role in the sensitization process. The obtained results may improve our understanding of the primary sensitization mechanisms with the involvement of the PR-10 family of lipid-binding allergens.

## 1. Introduction

The prevalence of allergic diseases including asthma is increasing worldwide, making it a major public health concern [1]. Pollen allergies affect approximately 10–30% of the global population, with higher rates observed in urbanized regions [2]. It has been estimated that 40% of Europeans are sensitized to pollen allergens [3].

Birch pollen is a major contributor to allergic rhinitis and asthma, affecting millions of people, especially in North America and in Central and Northern Europe [4]. The most relevant sensitizing protein for birch pollen-allergic individuals is Bet v 1 from the birch Betula verrucosa, belonging to the ubiquitous PR-10 protein family [5]. All known members of this protein family are able to bind a broad spectrum of hydrophobic molecules. Along with ligand-binding properties, they constitute one of the most relevant classes of plant panallergens, including dozens of plant food and pollen allergen proteins sharing similar spatial structures and causing severe allergic reactions [4]. The under-investigated PR-10 protein Aln g 1 from the alder *Alnus glutinosa* pollen is a clinically relevant allergen. It has been considered as a cross-reactive allergen causing allergic reactions in patients sensitized to the birch Bet v 1. However, recent studies demonstrated that Aln g 1 was able to cause Bet v 1-independent sensitization of the immune system, suggesting that allergen-specific immunotherapy with Bet v 1 for patients with birch-related allergy to alder might be ineffective [6].

Airway epithelial cells comprise the front line to encounter aeroallergens and, therefore have become a subject of many investigations. Recent studies have reported that epithelial cells play dual roles as a protective barrier and a sensor integrating environmental signals [7]. The first necessary step in the sensitization process is the penetration of an inhaled allergen through the respiratory epithelium. However, before interacting with respiratory epithelial cells, aeroallergens must overcome the surfactant barrier that overlays the human airways. The surfactant layer plays a crucial role in the innate immune defense of the airways and can influence the interaction between allergens and epithelial cells. To date, the mechanism of interaction of allergens with a surfactant has not been elucidated. The birch pollen allergen Bet v 1 has been demonstrated to move through nasal epithelium isolated from patients with the birch pollen allergy, probably via caveolae-dependent uptake and transport [8]. At the same time, there are no studies of the transepithelial transport of the alder pollen allergen Aln g 1 across either the human respiratory epithelium or the polarized airway epithelial cell monolayer.

The allergen transport through the epithelium stimulates epithelial cells to produce alarmins, including TSLP, IL-33, and IL-25, which subsequently activate the immune system’s allergic response through type 2 innate lymphoid cells (ILC2) [9,10]. Previously, we demonstrated that Aln g 1 induced the upregulation of the alarmins TSLP and IL33 genes in airway epithelial Calu-3 cells after a 24-h exposure period [11]. Other cytokines and chemokines may also participate in the development of allergic diseases. For instance, recently the pro-inflammatory IL-1β was demonstrated to be of great importance in the development of allergic diseases [12]. Also, it has been shown that epithelial cells could produce the most potent human neutrophil-attracting chemokine CXCL8 upon stimulation with allergens [13,14].

The main aim of this study was to investigate the major alder allergen Aln g 1 transport through the human airway epithelial barrier in comparison with that of the main sensitizer of the PR-10 class of allergens—the birch pollen allergen Bet v 1. For that aim, by using fluorescence spectroscopy, we performed a comparative study of the ability of the alder Aln g 1 and the birch Bet v 1 to interact with liposome membranes modeling the lipid composition of the adult human pulmonary surfactant. Then, we investigated bidirectional transport of Aln g 1 and Bet v 1 through the Calu-3 polarized monolayer as an in vitro model of the bronchial epithelial barrier. Moreover, by using qPCR, we investigated the relative gene expression of alarmin and other pro-inflammatory genes in epithelial cells in response to stimulation of the Calu-3 polarized monolayer with Aln g 1 and Bet v 1 allergens.

## 2. Results

### 2.1. Calcein-Leakage Induced by Allergens

In order to model the interaction of Aln g 1 and Bet v 1 with the surfactant layer of epithelial barrier we used calcein-containing small unilamellar vesicles. Owing to the self-quenching property, high concentrations of calcein inside the liposome determine its minimal fluorescence. However, once the dye is released from liposomes and highly diluted, the fluorescence signal tends to increase. Calcein leakage was used as a standard method for investigating membrane perturbations. The liposome lipid composition (DPPC:DOPC:DOPG:DOPE = 70:10:10:10) was selected based on the phospholipid composition of adult human pulmonary surfactant [15]. DPPC:DOPC:DOPG = 80:10:10 and DPPC:DOPC:DOPE = 80:10:10 liposomes were modeled to assess the contribution of specific lipids to allergen interactions with membranes.

Before studying the calcein leakage, the hydrodynamic diameter of the obtained liposomes was determined using the dynamic light scattering (DLS). DLS is a widely used technique, which offers rapid, non-destructive analysis of vesicle size distribution and stability [16]. Measurement results were shown in Table S1. For all samples hydrodynamic diameter was <150 nm and polydispersity index was <0.2.

**Figure 1:**
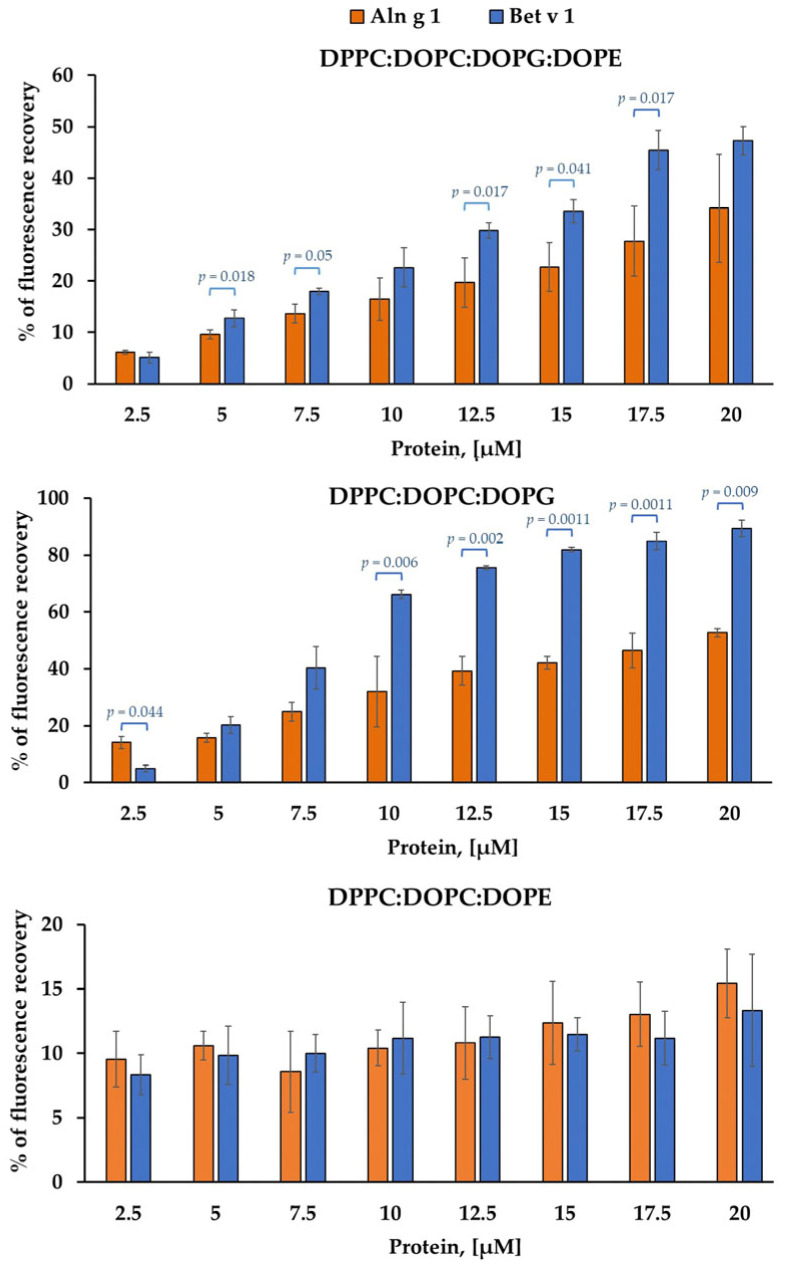
Percentage of calcein dye leakage from SUVs upon addition of different concentrations of Aln g 1 and Bet v 1. % of fluorescence recovery between allergens at the same concentration was compared by unpaired two-sample *t*-test. Data are represented as mean ± SD from three biological replicates (*n* = 3).

All tested liposomes exhibited leakage of calcein in response to allergens, with the degree of leakage varying according to the protein concentration. For DPPC:DOPC:DOPG:DOPE and DPPC:DOPC:DOPG vesicles, Aln g 1 was less effective than Bet v 1 (Figure 1). However, in the case of DPPC:DOPC:DOPE liposomes, Aln g 1 and Bet v 1 were found to release calcein from such liposomes with similar effectiveness (Figure 1). It is worth noting that dynamic light scattering experiments showed that, although the integrity of liposomes is disrupted due to interaction with allergens, their overall architecture is likely to be preserved.

### 2.2. Transport of Aln g 1 and Bet v 1 Across the Calu-3 Epithelial Barrier

Calu-3 is a human lung cancer cell line that is widely used as an in vitro model of pulmonary infection by viruses [17] and pulmonary permeability for drugs [17]. This line is one of the most useful cell lines for studying the bronchial epithelium, as this cell line growing in cell inserts as a polarized monolayer was found to have the closest resemblance to this epithelium in vivo [18]. Among several bronchial epithelial cell lines, commonly used in studies, only Calu-3 simultaneously displays the following properties: chloride (Cl^−^) secretion in a cAMP-dependent manner, expression of the cystic fibrosis transmembrane conductance regulator (CFTR), ability to form a polarized monolayer with apical microvilli and tight junctions between the cells, expression of the lung surfactant-specific protein proSP-C and production of mucin granules [19]. Moreover, it was previously shown that the permeability characteristics of Calu-3 cells correlated well with the rate of drug absorption from the rat lung in vivo [20].

**Figure 2:**
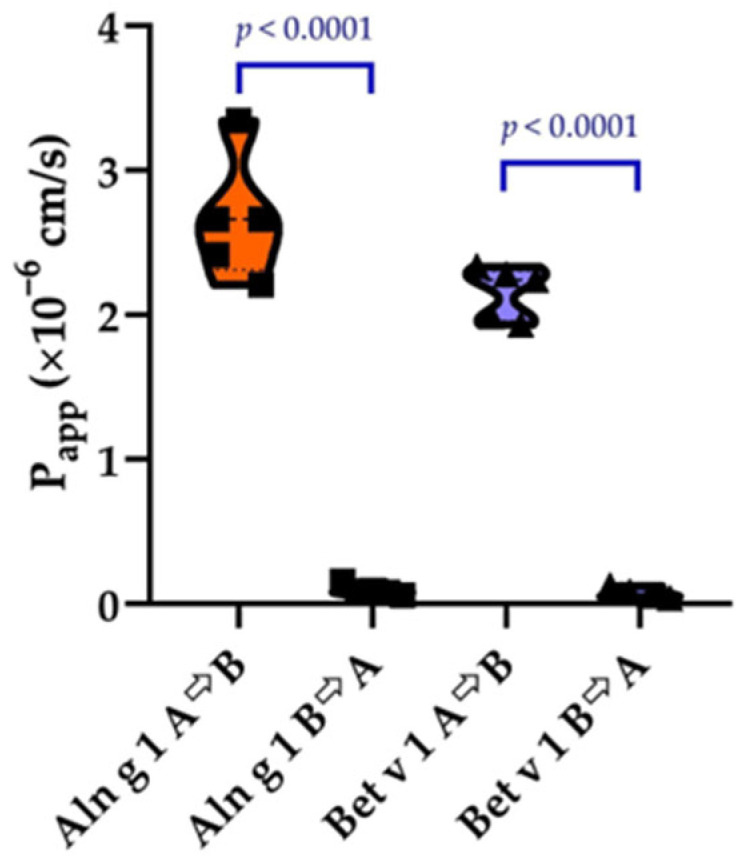
Assessment of bidirectional transport of alder allergen Aln g 1 (orange) and birch allergen Bet v 1 (blue) through the polarized Calu-3 monolayer. A→B, absorptive transport; B→A, secretory transport; P_app_—apparent permeability coefficient. The normality of P_app_ coefficient distribution was assessed using Shapiro–Wilk test. P_app_ coefficients were compared by unpaired two-sample *t*-test. Data are represented as mean ± SD from five biological replicates (*n* = 5).

For the first time we assessed the transport of PR-10 allergens through the polarized Calu-3 monolayer as a model of the pulmonary epithelium. The bidirectional transport of FITC-labelled Bet v 1 and Aln g 1 was studied (Figure 2). We demonstrated that “apical-to-basolateral” (A→B, absorptive) transport was much more effective than in the “basolateral-to-apical” (B→A, secretory) direction. The apparent permeability coefficients (P_app_) for both proteins were determined to be 2.66 ± 0.28 × 10^−6^ cm/s and 2.15 ± 0.16 × 10^−6^ cm/s at a 5 μM concentration in the donor chamber for Aln g 1 and Bet v 1, respectively. The transport in the secretory (B→A) direction was much lower with the apparent permeability coefficients for Aln g 1 and Bet v 1 being 9.24 ± 2.49 × 10^−8^ cm/s and 7.04 ± 2.7 × 10^−8^ cm/s, respectively. Uptake ratios (UR) for both allergens were calculated and estimated to be 29.7 ± 0.9 for both allergens. Based on high uptake ratios we suggest for both allergens an active transport through Calu-3 polarized monolayer. However, while the Calu-3 model provided reproducible and mechanistically informative data, it is important to note that these immortalized cells may not fully reproduce the complexity of primary human airway epithelium, particularly in the context of allergic sensitization. Future studies using primary nasal or bronchial epithelial cells from allergic donors would strengthen the translational relevance of these findings.

### 2.3. Influence on the Expression of Alarmin Genes in Epithelial Cells

Our next step was to investigate the response of the epithelial cells upon stimulation of the Calu-3 polarized monolayer with Aln g 1 and Bet v 1 during different time intervals. Different intervals were checked to analyze the cytokine response, including early (2 h and 6 h) and late (24 h). For this purpose, we used qPCR. The housekeeping genes glyceraldehyde-3-phosphate dehydrogenase (GAPDH) and actin-γ were chosen as reference genes to calculate relative expression levels. In this case, Cq normalized by the average primers efficiency in each case should be analyzed to determine the expression change more accurately. The average PCR efficiency for each primer pair was calculated on web-service RDML-LinRegPCR [21]. It is important to note that LinRegPCR itself slightly underestimates efficiency, while being one of the most accurate methods for analyzing qPCR data [22]. After recalculation, the 2^−ΔΔCq^ method was used, and the data obtained were similar to those obtained using the CFX Maestro software version 2.3 (Bio-Rad, Hercules, CA, USA) without taking into account the efficiency of gene amplification.

**Figure 3:**
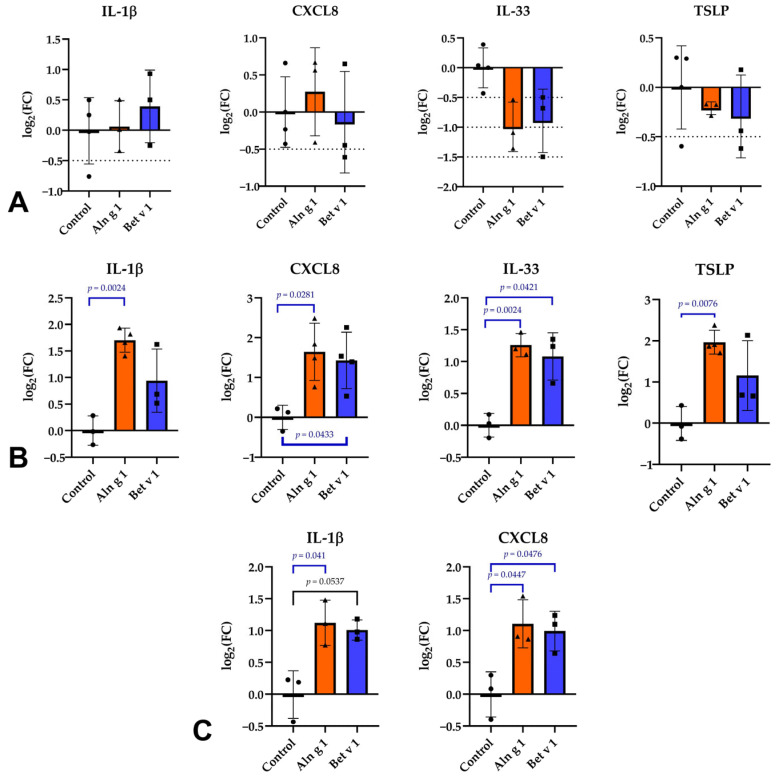
Analysis of relative gene expression levels of IL-33, TSLP, IL-1β and CXCL8 in Aln g 1- or Bet v 1-treated Calu-3 cells in 2 h (**A**), 6 h (**B**) and 24 h (**C**). Log2(Fold changes (FC = 2^−ΔΔCq^)) are presented as mean ± SD. mRNA levels measured upon treatment were compared to the corresponding non-treated samples, and the graph was prepared using GraphPad Prism v 8.0.1 (GraphPad Software, San Diego, CA, USA). Each biological replicate is demonstrated as different symbols: ●—control, ▲—Aln g 1-treated, ■—Bet v 1-treated. Biological replicates were included in triplicate or quadruplicate (*n* = 3–4).

For the assessment of the impact of the allergens to epithelial cells, the Calu-3 polarized monolayer was stimulated for 2, 6 or 24 h in a complete medium containing 5 μM of Aln g 1, Bet v 1, or in a medium alone as a control. As target genes IL25, IL33, TSLP, IL1B, and CXCL8 were chosen. Researchers have already shown increased expression of these cytokines by proteins belonging to various groups, including lipid-binding allergens [23,24,25,26,27]. Both in our previous work and in this study, we failed to evaluate IL25 gene expression at the mRNA level probably due to the low representation of its transcripts. This is similarly confirmed by this study, where the IL25 gene expression failed to be reliably detected in epithelial cells isolated from healthy individuals and patients with asthma [28]. We observed no statistically significant changes in gene expression during stimulation of the epithelial monolayers for 2 h (Figure 3A). However, after stimulation for 6 h, significant changes in the case of all cytokines were observed for Aln g 1. Interestingly, genes of IL-1β and TSLP were upregulated by stimulation with Bet v 1 to a lesser extent (Figure 3B). For long-term exposure of 24 h, we previously showed that Aln g 1 was able to induce IL33 and TSLP upregulation in the polarized Calu-3 monolayer [11]. In the current study we demonstrated that Aln g 1 and Bet v 1 pollen allergens are able to stimulate the expression of not only IL-33 and TSLP but also the pro-inflammatory cytokine IL-1β and neutrophil-attracting chemokine CXCL8 (Figure 3C). We showed that Bet v 1 and Aln g 1 induced comparable upregulation of IL33, TSLP, IL1B, and CXCL8 genes in the polarized Calu-3 monolayer, mimicking the bronchial epithelium; however, these changes in cytokines production by epithelial cells should be validated at the protein level (ELISA or Western blot) in future work.

## 3. Discussion

The sensitization process of the human immune system begins with the invasion of an allergen through the epithelial surfaces of the respiratory tract, gastrointestinal tract or skin. When inhaled, pollen grains release a hydrophilic cocktail consisting of various proteins, lipids, carbohydrates and other compounds upon contact with the mucosal surface [29,30]. Due to their proteolytic, lipid-binding and microbe-mimicking properties, allergens can penetrate the mucous membrane of the respiratory tract and reach antigen-presenting cells (APCs) lying beneath the epithelium [31].

Firstly, aeroallergens encounter a surfactant, a complex mixture, predominantly composed of lipids (approximately 90% by weight), with the remaining 8–10% consisting of proteins. The lipidic fraction is characterized by a high proportion of phospholipids (80–85% by weight), accompanied by a minor component of neutral lipids (5–10%) [32].

Birch Bet v 1 and other PR-10 proteins are known to be able to bind a wide range of different ligands, including flavonoids, fatty acids (FAs) and their derivatives, cytokinins, and other molecules, for example, sphingosine [4]. We have previously shown that alder Aln g 1 is also capable of binding various fatty acids, lysolipids and phytosphingosine [11]. For Bet v 1, it is known that ligand binding can promote its potential translocation across membranes [33]. However, to date, there are no data demonstrating the ability of Aln g 1 to bind to and/or permeabilize membranes.

In the current work we studied for the first time the interaction of Aln g 1 and Bet v 1 with liposomes modeling the composition of a surfactant. Phosphatidylcholines make up approximately 75% of the phospholipids in pulmonary surfactant, together with dipalmitoylphosphatidylcholine (DPPC) being crucial for maintaining low surface tension at the alveolar air-liquid interface during expiration [32]. Negatively charged phosphatidylglycerol (8% by weight) and phosphatidylinositol (3% by weight) interact with cationic hydrophobic proteins, which facilitate the transfer of lipids to the interface and the subsequent formation of the surfactant film there, thereby contributing to the overall functionality of the surfactant complex [32,34,35,36]. Additionally, phosphatidylethanolamine (PE) (1.5%) promotes negative curvature in the lipid bilayer during respiration. Together these lipids are considered to be crucial for effective pulmonary surfactant function [37].

Based on the data on the lipid composition of the pulmonary surfactant, liposomes of DPPC/DOPC/DOPG/DOPE were created. It was established that both allergens can interact with the liposomes of this composition, inducing their damage. However, Bet v 1 causes calcein leakage from the liposomes more efficiently than Aln g 1. This process is probably affected by the structural features of proteins, charge distribution on their surface and electrostatic attraction and curvature of vesicles (and, consequently, surface cracks or defects). It is worth noting that the spatial structure of Aln g 1 has not yet been resolved. Next, we made an attempt to determine which component affects the destruction of liposomes. We found that the exclusion of DOPG from the lipid composition reduces the ability of allergens to cause dye leakage. It is interesting to note that at an allergen concentration of 2.5 μM, there is practically no dependence on the lipid composition of liposomes, but the ability to destroy membranes is preserved. Thus, it can be assumed that both allergens are able to disrupt the barrier layer of the surfactant and penetrate the epithelial layer.

The next barrier for allergens to penetrate after the surfactant is epithelial cells. There are no reports about Aln g 1 transport through the bronchial epithelium or its cellular models. And the only two articles available in the literature on the transport of PR-10 allergens across the airway epithelium are devoted to the transport of Bet v 1 through nasal and conjunctival epithelia [8,38]. In the first study the authors demonstrated transport through the nasal epithelium isolated from atopic patients and control individuals and suggested that Bet v 1 is transported by active caveolae/lipid raft-dependent uptake and subsequent transport [8]. In the second study, the same authors showed transport through the conjunctival epithelium obtained from healthy and allergic subjects. In both studies, the authors showed that the uptake and transport of the allergen through epithelia isolated from allergic patients were much more effective than ones from healthy individuals [38]. By using mice models of allergy for other allergens it was shown that they can rapidly be absorbed and transported through the respiratory epithelium in both sensitized and normal animals. For instance, Hens G. et al. applied ovalbumin (OVA) in the nose or trachea of sensitized and normal BALB/c mice and measured the serum OVA concentration [39]. The authors reported rapid systemic uptake of the allergen through the respiratory mucosa, as they detected OVA in serum in 15 min in both OVA-sensitized mice and normal BALB/c mice. In the current study, we reported the bidirectional transport of major pollen allergens, Aln g 1 and Bet v 1, through the polarized Calu-3 monolayer, mimicking the bronchial epithelial barrier. The Calu-3 cell line imposes certain limitations in terms of physiological relevance compared to primary epithelial cells; however, our aim was to establish a reproducible and widely accepted in vitro model to study the transepithelial transport of Aln g 1. Based on high uptake ratios for both allergens (>20), we proposed active transport of the allergens through the Calu-3 polarized monolayer. Our results coincide with previously obtained data that Bet v 1 allergen is probably subjected to active transport [8]. We demonstrated a similar penetration rate for Aln g 1 and Bet v 1 allergens, suggesting that the tremendous sensitization potential of Bet v 1 is likely not determined by its abnormal rate of transport through epithelium, which would sharply distinguish this allergen from other PR-10 allergens.

The encounter of the allergen with epithelial cells results in the stimulation of the latter and production of host defense peptides, cytokines and chemokines [40]. The cytokine environment created by epithelial cells is widely regarded as the “master switch” in the development of allergic diseases [40]. Allergens of a protein nature belonging to different non-protease groups are able to initiate an interaction-mediated epithelial release of cytokines, which can be directly related to the Th2-type response [23,24,26,27,41,42]. For example, it is well known that in response to tissue damage, pathogen recognition or allergen exposure the upper airway epithelium is able to secrete the alarmins TSLP, IL-33, and IL-25 [9].

The upregulation of these alarmins genes in epithelial cells was previously shown for peach Pru p 3, the major allergen of the class of lipid transfer proteins (LTPs) [24], and peanut Ara h 3, belonging to the class of 11S globulins [23]. We have also previously shown the ability of Aln g 1 to induce the gene expression of IL-33 and TSLP after stimulation for 24 h [11]. The latest studies report that other cytokines and chemokines can also participate in the development of allergic diseases. For instance, some recent data suggest that pro-inflammatory IL-1β may be of great importance in the development of allergic diseases and allergic inflammation [12]. Also, there are reports in the literature that epithelial cells can produce the most potent human neutrophil-attracting chemokine CXCL8 upon stimulation with allergens [13,14]. In the current study we decided to compare an epithelial response upon stimulation of the polarized Calu-3 monolayer with two major allergens, belonging to the PR-10 class—Bet v 1 from birch pollen and Aln g 1 from alder pollen. To do that, we assessed the gene expression of *TSLP*, *IL33*, *IL1B* and *CXCL8* genes after stimulation with the allergens during different time intervals. There were no significant changes in gene expression of all studied transcripts in the epithelial cells after stimulation by both allergens over 2 h (Figure 3A). Instead, Aln g 1 and Bet v 1 demonstrated an increase in expression levels following 6 and 24 h, with Bet v 1 displaying a lesser response in the case of TSLP and IL-1β. It is worth noting that we did not observe crucial differences in gene expression in the epithelial cells upon stimulation by birch pollen Bet v 1 and alder pollen Aln g 1 allergens. In summary, the data we obtained demonstrate that the transport of Aln g 1 and Bet v 1 pollen allergens through the epithelial barrier induces the pro-inflammatory cytokines and alarmins expression by epithelial cells, which probably contributes to the sensitization.

## 4. Materials and Methods

### 4.1. Materials

All lipids were purchased from Avanti Polar Lipids (Alabaster, AL, USA). The recombinant proteins were overexpressed in *Escherichia coli* and purified as described previously [11]. Homogeneity and the identity of the recombinant allergens were confirmed by SDS-PAGE and MALDI mass spectrometry (Supporting Materials Figures S1 and S2).

### 4.2. Human Cell Lines and Cultures

Calu-3, human lung adenocarcinoma line (ATCC HTB-55), was cultured in DMEM/F12 (Corning, Manassas, VA, USA) supplemented with 10% heat-inactivated FBS (Capricorn Scientific, Ebsdorfergrund, Germany), 1× antibiotic-antimycotic (Gibco, Waltham, MA, USA). Before the experiments the cells were subcultivated at least twice. For stimulation with proteins the cells were seeded in the wells of a 24-well plate at a density of 2.6 × 10^5^ cells/cm^2^ and cultured in a humidified CO_2_ incubator (5% CO_2_, 37 °C), changing the medium twice a week until reaching a monolayer.

For growing barriers mimicking bronchial epithelium, the Calu-3 cells were seeded onto cell culture inserts (0.4 μm, 0.6 cm^2^ surface area) (SPL Life Sciences, Pochon, Kyonggi-do, Republic of Korea) in the wells of a 24-well plate at a density of 7 × 10^5^ cells/cm^2^ and cultured in a humidified CO_2_ incubator (5% CO_2_, 37 °C), changing the medium every 2–3 days until polarized monolayer appeared. A transepithelial electrical resistance (TEER) was measured using Millicell ERS-2 Voltohmmeter (Merck-Millipore, Burlington, MA, USA) to test the polarization and integrity of the monolayer. Only inserts with TEER > 600 Ω cm^2^ were used in the transport assay.

### 4.3. Stimulation of Calu-3 Cells with Aln g 1 and Bet v 1

Calu-3 cells were stimulated with 5 μM Aln g 1 or Bet v 1 for different stimulation times (2, 6, or 24 h). Prior to stimulation, the culture medium was replaced with serum-free medium 24 h before the experiment and maintained in a humidified CO_2_ incubator (5% CO_2_, 37 °C). For each condition, three to four independent biological replicates were analyzed. Medium alone (without allergens) was used as the negative control. At the designated time points (2, 6, or 24 h), cells were lysed using ExtractRNA reagent (Evrogen, Moscow, Russia) and immediately frozen at −70 °C for subsequent RNA isolation.

### 4.4. RNA Extraction

Total RNA was isolated from the frozen samples following the manufacturer’s protocol using ExtractRNA reagent. Independent biological replicates were processed on separate days. RNA integrity was assessed by agarose gel electrophoresis, while RNA concentration and purity were determined via UV/Vis spectrophotometry using a Nanophotometer NP50 (Implen Inc., Westlake Village, CA, USA). All RNA samples demonstrated OD260/280 ratios > 1.8 and OD260/230 ratios > 2, confirming high purity.

### 4.5. Real-Time PCR

Real-time PCR was performed according to [11] with minor changes. Briefly, following RNA extraction and purification, 1 µg of total RNA was reverse-transcribed into cDNA using the MMLV Reverse Transcriptase kit (Eurogen, Moscow, Russia) according to the manufacturer’s protocol. The synthesized cDNA was then used to quantify relative gene expression. For qPCR, 12.5 ng of cDNA per reaction was utilized with qPCRmix-HS SYBR master mix (Eurogen), along with gene-specific primers (Table S2, Supporting Materials) at a final concentration of 0.5 µM, in a total reaction volume of 10 µL. Reactions were performed on a CFX Opus 96 Real-Time PCR System (Bio-Rad, Hercules, CA, USA). The thermal cycling conditions for qPCR amplification were as follows: the PCR was initiated by heating at 95 °C for 3 min; then 50 cycles of 95 °C for 15 s (denaturation), 60 °C for 20 s (annealing), and 72 °C for 30 s (extension). Following qPCR, melting curve analysis was conducted using a temperature increment (50–95 °C, increasing by 0.5 °C every 5 s). Reactions were performed in technical triplicates, and results were averaged across independent biological replicates. Primer efficiencies for each gene were evaluated using the RDML-LinRegPCR online tool 3.0.0 (https://www.gear-genomics.com/rdml-tools/linregpcr.html, accessed on 1 March 2025), with efficiencies falling within the 95–105% range. The mean primer efficiency was applied to compute normalized Cq values. Amplification specificity for the target genes was confirmed through melting curve analysis and agarose gel electrophoresis. For qPCR data analysis, the 2^−ΔΔCq^ method [43] was used.

### 4.6. Preparation of Calcein-Loaded Liposomes and Leakage Experiments

For making the calcein-entrapped small unilamellar vesicles (SUVs) composed of DPPC:DOPC:DOPG:DOPE, DPPC:DOPC:DOPG, DPPC:DOPC:DOPE a lipid film was rehydrated with PBS buffer containing 80 mM of calcein, pH 7.4. The liposomal suspension underwent ten cycles of freezing and thawing, and was subsequently extruded ten times through a polycarbonate filter with a pore size of 100 nm. Unentrapped calcein was eliminated via gel filtration using a Sepharose CL-4B (GE Healthcare, Chicago, IL, USA) column. The eluted calcein-entrapped vesicles were diluted to achieve the desired lipid concentration [44]. Prior to further experimentation, the fluorescence emission differences between the harvested vesicles and those subjected to collapse were evaluated. Fluorescence measurements were conducted at 20 °C using a F-2710 spectrofluorometer (Hitachi High Technologies America Inc., Pleasanton, CA, USA), with emission and excitation wavelengths set at 535 nm and 485 nm, respectively. Only liposomes demonstrating a minimum of a 5-fold difference between assembled and disrupted forms were used in subsequent studies.

An assay on the ability of proteins to permeabilize the membrane was conducted in three replicates on a Plate Reader AF2200 (Eppendorf, Hamburg, Germany) at a wavelength of extinction and emission at 485 and 535, respectively. A 1% Triton X-100 solution was utilized as a positive control for membrane degradation. The percentage of dye leakage was quantified using the equation: dye leakage (%) = (F − F0)/(Ft − F0) × 100%, where F0 denotes the fluorescence intensity prior to protein addition, and F signifies the intensity following the addition of proteins. Each experiment was performed independently in sets of three. Data are presented as means ± SD, calculated across all treatments using GraphPad Prism, with significant differences assessed via *t*-test.

### 4.7. Dynamic Light Scattering

The mean hydrodynamic diameter of SUVs was measured via dynamic light scattering (DLS) using a particle size analyzer Litesizer 500 (Anton Paar GmbH, Graz, Austria). All samples were diluted in PBS by a factor of 1:85. All determinations were made at 25 °C with a light incidence angle of 90°. The hydrodynamic diameter followed a Gaussian distribution and the polydispersity index was determined according to the width of particle size distribution. Major parameters included hydrodynamic diameter (<150 nm) and polydispersity index (<0.2).

### 4.8. Labeling of Aln g 1 with FITC

Recombinant Aln g 1 and Bet v 1 were conjugated with fluorescein isothiocyanate isomer I (FITC) (Sigma-Aldrich, St. Louis, MO, USA). Briefly, 1.7 mg of each protein was dissolved in 50 µL dimethyl sulfoxide (DMSO), mixed with 300 µL coupling buffer (0.1 M sodium carbonate-bicarbonate, pH 10.0), and combined with 3.5 mg FITC dissolved in 100 µL DMSO. The labeling reaction was incubated for 2 h at 20 °C under light-protected conditions to prevent fluorophore degradation. The separation of FITC bound to proteins from unbound FITC was performed using PD-10 column (GE Healthcare, Chicago, IL, USA) previously equilibrated with transport buffer (Hank’s balanced salt solution, containing 25 mM HEPES, 1 mM MgCl_2_, 1 mM CaCl_2_, and 10 mM D(+)glucose, pH 7.4). The solution of FITC-labeled protein in the transport buffer was used in transport assay.

### 4.9. Transport of FITC-Aln g 1 Across the Calu-3 Epithelial Barrier

The penetration capability of FITC-labelled Aln g 1 or Bet v 1 was investigated using Calu-3 cells as an in vitro model bronchial epithelial barrier, grown on permeable cell culture inserts. Transport of the compound from both sides of the monolayer was determined in the transport buffer (Hank’s balanced salt solution, containing 25 mM HEPES, 1 mM MgCl_2_, 1 mM CaCl_2_, and 10 mM D(+)glucose, pH 7.4). To study the transport in the “apical-to-basolateral” (A→B) direction, 0.7 mL of the transport buffer (pH 7.4) was added to the wells of a 24-well plate, and 5 µM compound in the same transfer buffer was added to the apical chamber. To study the transport in the “basolateral-to-apical” (B→A) direction, 0.4 mL of the transport buffer was added to the apical chamber, while 5 µM compound in 0.7 mL of the same transfer buffer was added to the wells of the 24-well plate from the basolateral side of the monolayer. The transfer buffer and the donor solution (5 µM of Aln g 1 or Bet v 1 in the transfer buffer) were pre-heated to 37 °C prior to the experiment. Transport of Aln g 1 or Bet v 1 through Calu-3 polarized monolayers was conducted for 90 min in 5 independent inserts for each direction.

Apparent permeability coefficients (P_app_) for each compound under steady-state conditions for each direction and each insert were calculated according to Equation (1):

P_app_ = ΔC/Δt × (V/(A × Ci)), (1)

where ΔC—protein concentration in acceptor chamber (μM), Δt—incubation period (s), V—volume of the solution in acceptor chamber (ml), A—an area of the insert (cm^2^), Ci—initial concentration (μM). In order to verify the monolayer integrity, the apparent permeability of a paracellular marker, Lucifer Yellow (Sigma), was estimated. For the calculation of uptake ratio (UR), which is forward to reverse transport ratio, the following equation was used (2):

UR = P_app_ (A→B)/P_app_ (B→A). (2)

The integrity of the monolayers was checked by measuring TEER at the beginning and after the end of the transport experiment.

### 4.10. Statistical Analysis

Data were analyzed using Graphpad Prism 8.0.1. Differences between groups in qPCR were analyzed using Welch and Brown-Forsythe ANOVA. The normality of apparent permeability coefficient (P_app_) distribution was assessed using Shapiro–Wilk test. Papp coefficients were compared using unpaired two-sample *t*-test. Values were considered significantly different when *p* < 0.05. Data on the liposome leakage are presented as means ± SD, with significant differences assessed via *t*-test.

## 5. Conclusions

The lipid-binding allergens Aln g 1 from the alder pollen and Bet v 1 from the birch pollen are two clinically relevant allergens, belonging to the PR-10 class. The sensitization of the immune system to pollen allergens is believed to begin from the inhaled allergen transport through to the respiratory epithelium and contact with antigen-presenting cells. This study provides new insights into the early stages of allergic sensitization by comparing alder Aln g 1 and birch Bet v 1. Our key findings demonstrate that both allergens disrupt pulmonary surfactant-mimicking liposomes, but Bet v 1 exhibits significantly stronger membrane-permeabilizing activity, and negatively charged phosphatidylglycerol (DOPG) enhances this effect, suggesting electrostatic interactions contribute to membrane disruption. Using a polarized Calu-3 monolayer, we established, for the first time that Aln g 1 undergoes transcellular transport at rates comparable to Bet v 1, and uptake ratios for both allergens exceeded 20; supporting the hypothesis of active transport of the allergens through the Calu-3 polarized monolayer. We showed that Aln g 1 upregulates Th2-promoting alarmins (IL-33, TSLP) and pro-inflammatory mediators (IL-1β, CXCL8) in epithelial cells, with Bet v 1 showing a weaker but significant response. This suggests Aln g 1 may initiate stronger innate immune activation, despite similar transport kinetics. The observed differences in membrane disruption efficiency but comparable transcytosis rates and diverse cytokine induction profiles suggest that the sensitization potential is not solely determined by epithelial penetration speed and that structural features of allergens (e.g., ligand-binding pockets, surface charge distribution) may have an impact on allergen-epithelium interactions. Together, our findings provide a foundation for further research to explore the specific mechanisms by which allergen structural features influence sensitization, paving the way for the development of targeted allergy therapies.

## Data Availability

All data generated and analyzed during this study are included in this published article and its Supplementary Materials file.

## Supplementary Materials

The following supporting information can be downloaded at: https://www.mdpi.com/article/10.3390/ijms26115169/s1.

## Abbreviations

The following abbreviations are used in this manuscript:

DPPC	1,2-dipalmitoyl-sn-glycero-3-phosphatidylcholine
DOPC	1,2-dioleoyl-sn-glycero-3-phosphocholine
DOPG	1,2-dioleoyl-sn-glycero-3-phosphoglycerol
DOPE	1,2-dioleoyl-sn-glycero-3-phosphoethanolamine
LTPs	Lipid transfer proteins
OVA	Ovalbumin
SUVs	Small unilamellar vesicles
TEER	Transepithelial electrical resistance
