# Supplementary material for: Modeling Human Airway Epithelial Barrier Penetration Using Birch Bet v 1 and Alder Aln g 1 Pollen Allergens During Sensitization Process
Source: Int J Mol Sci. 2025 May 28;26(11):5169. doi: 10.3390/ijms26115169 (PMC12155471; doi:10.3390/ijms26115169)
Supplement: Supplementary file 1 [file ijms-26-05169-s001.zip › ijms-3567012-supplementary.pdf]

Supplementary Materials

Modeling the human airway epithelial barrier penetration by the birch Bet v 1 and the alder Aln g 1 pollen allergens during sensitization process

Daria N. Melnikova [1,2,*,†], Andrey E. Potapov [1,2,†], Tatiana V. Ovchinnikova [1,2] and Ivan V. Bogdanov [1]

[1] M.M. Shemyakin & Yu.A. Ovchinnikov Institute of Bioorganic Chemistry, the Russian Academy of Sciences, 117997 Moscow, Russia; d_n_m_@mail.ru (D.N.M.); cool.goyan@yandex.ru (A.E.P.); ovch@ibch.ru (T.V.O.); contraton@mail.ru (I.V.B.)
[2] Moscow Center for Advanced Studies, 123592 Moscow, Russia
[*] Correspondence: d_n_m_@mail.ru; Tel.: +7-495-335-42-00
[†] These authors contributed equally to this work.

Table S1. Hydrodynamic diameter and polydispersity index of liposomes

Samples	Hydrodynamic diameter, nm	Polydispersity index
DPPC:DOPC:DOPG:DOPE	141.05 ± 5.6	0.021±0.02
DPPC:DOPC:DOPE	141.14 ± 3.2	0.114 ± 0.03
DPPC:DOPC:DOPG	125.07 ± 6.4	0.102 ± 0.02

Table S2. Primer sequences for real-time PCR.

Name	Sequence (5′→ 3′)	Notes (the amplicon length)
TSLP-F	GCTATCTGGTGCCCAGGCTAT	Sense primer to TSLP (131 b.p.)
TSLP-R	CGACGCCACAATCCTTGTAAT	Anti-sense primer to TSLP (131 b.p.)
IL-33-F	CACCCCTCAAATGAATCAGG	Sense primer to IL-33 (115 b.p.)
IL-33-R	GGAGCTCCACAGAGTGTTCC	Anti-sense primer to IL-33 (115 b.p.)
IL-1β-F	CGATGCACCTGTACGATCAC	Sense primer to IL-1β (250 b.p.)
IL-1β-R	CAGCTGTAGAGTGGGCTTATC	Anti-sense primer to IL-1β (250 b.p.)
CXCL8-F	CTTGGCAGCCTTCCTGATTT	Sense primer to CXCL8 (249 b.p.)
CXCL8-R	AACTTCTCCACAACCCTCTG	Anti-sense primer to CXCL8 (249 b.p.)
GAPDH-1F	GGGGAGCCAAAAGGGTCATCATCT	Sense primer to GAPDH (235 b.p.)
GAPDH-1R	GAGGGGCCATCCACAGTCTTCT	Anti-sense primer to GAPDH (235 b.p.)
ACT-γ-F	AGGCCAACAGAGAGAAGATGACT	Sense primer to actin-γ (135 b.p.)

ACT-γ-R	CGTCTCCAGAGTCCATGACA	Anti-sense primer to actin-γ (135 b.p.)

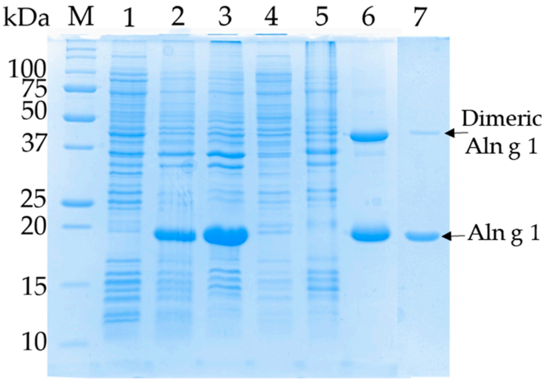

Figure S1. Electrophoretic analysis of the isolation and purification steps of rAln g 1 (rBet v 1 is identically). M - marker; 1 - total cell lysate without induction; 2 - total cell lysate after autoinduction with lactose; 3 - inclusion bodies; 4 - soluble cellular protein fraction; 5 – slug; 6 - eluate; 7 - rAln g 1 after RP-HPLC

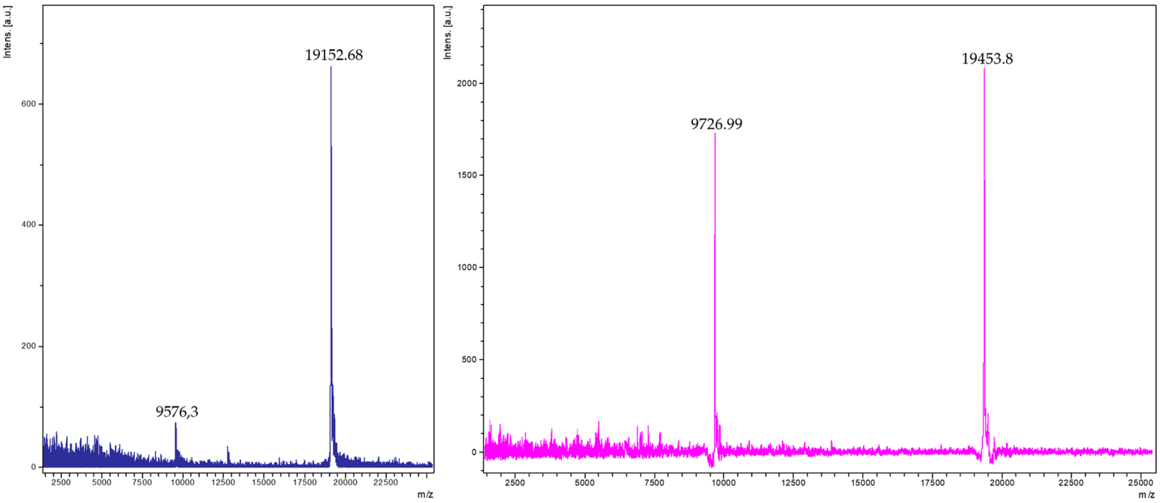

Figure S2. MALDI mass spectrometry rAln g 1 and rBet v 1.
